# Bioinformatis analysis reveals possible molecular mechanism of PXR on regulating ulcerative colitis

**DOI:** 10.1038/s41598-021-83742-8

**Published:** 2021-03-08

**Authors:** Hanze Guo, Yan Chi, Naiyu Chi

**Affiliations:** 1 College of Life Sciences, Liaoning Normal University, Dalian, 116081 China; 2 College of Life Science and Technology, Dalian University, Dalian, 116622 China

**Keywords:** Biochemistry, Computational biology and bioinformatics

## Abstract

Inflammatory bowel disease (IBD) is a chronic, recurrent inflammatory disease of the gastrointestinal (GI) tract. Ulcerative colitis (UC) is a type of IBD. Pregnane X Receptor (PXR) is a member of the nuclear receptor superfamily. In order to deepen understanding and exploration of the molecular mechanism of regulation roles of PXR on UC, biological informatics analysis was performed. First, 878 overlapping differentially expressed genes (DEGs) between UC and normal samples were obtained from the Gene Expression Omnibus (GEO) database (GSE59071 and GSE38713) by using the "limma" R language package. Then WGCNA analysis was performed by 878 DEGs to obtain co-expression modules that were positively and negatively correlated with clinical traits. GSEA analysis of PXR results obtained the signal pathways enriched in the PXR high and low expression group and the active genes of each signal pathway. Then the association of PXR with genes that are both active in high expression group and negatively related to diseases (gene set 1), or both active in low expression group and negatively related to diseases (gene set 2) was analyzed by String database. Finally, carboxylesterase 2 (CES2), ATP binding cassette subfamily G member 2 (ABCG2), phosphoenolpyruvate carboxykinase (PCK1), PPARG coactivator 1 alpha (PPARGC1A), cytochrome P450 family 2 subfamily B member 6 (CYP2B6) from gene set 1 and C-X-C motif chemokine ligand 8 (CXCL8) from gene set 2 were screened out. After the above analysis and reverse transcriptase quantitative polymerase chain reaction (RT-qPCR) verification, we speculated that PXR may exert a protective role on UC by promoting CES2, ABCG2, PCK1, PPARGC1A, CYP2B6 expression and inhibiting CXCL8 expression in their corresponding signal pathway in intestinal tissue.

## Introduction

Inflammatory bowel disease (IBD) is a chronic, recurrent inflammatory disease of the gastrointestinal (GI) tract^1,2^. Ulcerative colitis (UC) is known as one type of IBD^3^. The incidence of UC is increasing not only in Western countries, but also in developing countries. Lesions in UC are mostly located in colon and rectum, which can further spread to the whole colon and attack repeatedly^4^. The clinical features of UC mainly manifest as diarrhea, abdominal pain, mucus defection, bloody stool and weight loss, etc. The etiology of UC is not yet very clear. At present, it is believed that the intricate coordination between genetics, environment, and immunity affects the occurrence and development of diseases^5,6^. In the past 50 years, many advances have been made in the treatment of UC. Biological agents, biosimilars, and antibiotics et al^7,8^, all have certain therapeutic effects on UC. Among them, rifaximin is an oral antibiotic that is virtually unabsorbed and only acts locally in the intestinal tract, and is thus with low systemic response. Many studies have confirmed its therapeutic effect and safety^9,10^.

Rifaximin is an agonist of nuclear receptor (NR) Pregnane X receptor (PXR)^11^. NR is a type of ligand activated transcription factor. It regulates gene expression by inhibiting the binding of repressor proteins and recruiting co-activators. In recent years, more and more studies have shown that NRs play an important role in maintaining intestinal nutrient absorption and transport, intestinal barrier function, and intestinal immunity^12^. PXR is a member of the NR superfamily and is highly expressed in liver and intestinal tissues. In recent years, it has been reported that PXR can inhibit the development of UC by regulating the xenobiotics metabolism, anti-inflammatory, and maintaining the barrier function of intestinal epithelial cells^13,14^, indicating that using PXR as a drug target to treat UC has certain application prospects. However, the pathological process of UC is very complicated. Metabolic abnormalities^15^, inflammation, immune response, cell proliferation, and apoptosis et al. are all involved in UC^16,17^. With the increase in research on PXR, the physiological role of PXR has also been better understood. In addition to acting as a sensor for exogenous substances detoxification, drug metabolism, and drug interactions by regulating the expression of enzyme encoding drug metabolism or drug transporter genes, PXR also plays an important regulatory role in glycolipid metabolism^18^, inflammation, cell proliferation, apoptosis, cell migration, and immune regulation^19–21^. However, whether PXR could regulate UC through these roles is not completely clear. Therefore, deeply understanding and exploration of the molecular mechanism of regulation roles of PXR on UC are of great significance for the treatment of UC as PXR being a drug target in the future.

Bioinformatics can effectively analyze gene chip data. As the latest bioinformatics research method, weighted gene co-expression network analysis (WGCNA) is a system biology method that uses gene expression data to construct a scale-free network. It can cluster genes with similar expression patterns into the same gene module. By associating modules with external sample traits, the relationship between modules and phenotypes could be explored^22^. Gene Set Enrichment Analyses (GSEA) could reveal the biological signal pathway by enriching genes into related gene sets^23^.

In this study, WGCNA was used to cluster DGEs into co-expression modules and correlate module with clinical traits. Then GSEA was performed to obtain the signaling pathways and corresponding active genes enriched in PXR high and low expression group. Based on the above analysis, the protein–protein interaction (PPI) network was carried out to screen and identify genes that are interacted and functionally associated with PXR. At last, reverse transcriptase quantitative polymerase chain reaction (RT-qPCR) was performed to verify the results of bioinfomatics analysis. The research provides data support for exploring the molecular mechanism of PXR regulating UC.

## Methods

### Data information

The gene expression profile of GSE59071 and GSE38713 was obtained from the GEO database (https://www.ncbi.nlm.nih.gov/geo/). GSE59071, the platform is the [HuGene-1_0-st] Affymetrix Human Gene 1.0 ST Array, which includes 74 UC samples and 11 normal. GSE38713, the platform is the [HG-U133_Plus_2] Affymetrix Human Genome U133 Plus 2.0 Array, consists of 15 UC samples and 13 normal samples.

### Data preprocessing and DEGs analysis

**Figure 1:**
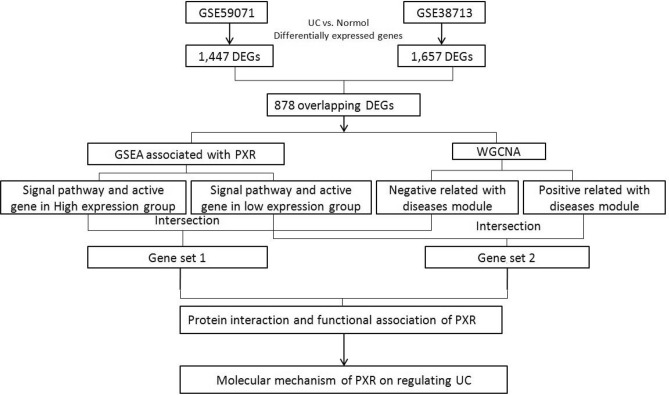
Flow diagram of study.

The research was designed according to the flow chart (Fig. 1). There were a total of 20,741 probes in GSE59071 datasets and 21,753 probes in GSE38713 datasets, which are all annotated by the Affymetrix annotation file. Log2 transformation and normalization were performed for processing data. Based on the initial data, we utilized the "limma" R package in R to screen the DEGs between UC samples and normal samples. Genes with adjusted *P*-value (adj. *P*) < 0.05 and |log Fold Change| (|log FC|) > 0.9 were considered as DEGs.

### WGCNA analysis

We performed WGCNA analysis by WGCNA R package in R. WGCNA is a systematic biological method for constructing scale-free networks using gene expression data. Firstly the expression data profile of the overlapping DEGs in GSE59071 was tested to see whether they were suitable samples and genes. And then the similarity matrix was established according to Pearson’s correlation. Thirdly, the adjacency matrix was constructed to obtain a scale-free network from the similarity matrix by computing a correlation raised to a soft threshold power β between every pair of genes. Fourthly, the adjacency matrix was transformed into a topological overlap matrix (TOM) and the corresponding dissimilarity (dissTOM) was then calculated. Hierarchical clustering based on dissTOM was carried out to get a systematic clustering tree. With the parameters of the minimum number of base 30, the dynamic tree cut algorithm groups genes into modules. After that, according to the cutoff value of 0.25, module eigengenes (MEs) were clustered in order to merge very similar modules into a new one. We also calculated the correlation between the clinical traits and ME in each module.

### GSEA analysis

GSEA is a computational approach to identify significantly enriched or depleted groups of genes^23,24^. We used GSEA 4.0.3 software for our analysis. The median expression value of PXR was used as the cut-off value. 74 UC samples from the GSE59071 GEO database were divided into high expression group and low expression group. GSEA was conducted to analyze biological pathways associated with PXR. Gene size ≥ 5, |normalized enrichment score (NES)|> 1.0, and the top 20 pathway were used as the cut-off criteria.

### Protein interaction and functional association of PXR

STRING (version 11.0) database (https://string-db.org)^25^ was used to build the PPI network (score > 0.4) and the protein that interacted and associated with PXR was screened out. Cytoscape software was used for visualization^26^.

### Identification of PXR target genes

Pearson’s correlation coefficients (R-values) between the expression levels of PXR and the other genes were analyzed by the "Pearson" method in R Studio. JASPAR (http://JASPAR.genereg.net/) database contains a curated, non-redundant set of profiles, derived from published and experimentally defined transcription factor binding sites for eukaryotes^27^. The PXR binding site on the target gene promoter was predicted by the JASPAR database.

### Cell culture and adenovirus infection

HCT116, epethelial-like cell isolated from colon carcinoma, were cultured in DMEM containing 10% FBS with penicillin (100 U/ml) and streptomycin (100 U/ml) at 37 °C in a 5% CO_2_ incubator. In order to increase the expression level of PXR in HCT116, adenovirus PXR (Ad-PXR) was used to infect HCT116 for 24 h.

### RT-qPCR

Total RNA was isolated using TRIzol (Sangon), converted to complementary DNA (cDNA) by HiScript III 1st Strand cDNA Synthesis Kit (Vazyme). RT-qPCR was performed by using ChamQ® Universal SYBR qPCR Master Mix (Vazyme). Primer sequences were described in supplementary Table 1. Amplification was carried out with an initial step at 95 °C for 30 s, followed by 40 cycles of amplification (95 °C for 10 s, 60 °C for 30 s) by using a CFX96 qPCR system (Bio-Rad). GAPDH was used as an internal control, and data were expressed as the ratio of target mRNA to GAPDH mRNA. All results were representative of at least three independent experiments.

**Table 1:** Biological signal pathways and active genes in PXR high expression group by GSEA.

Pathway regulated by PXR	NES	Size	Active genes
PEROXISOME	1.32	13	EPHX2 XDH EHHADH PEX11A PHYH PEX26 PXMP2 ABCD3 ACOX1 NOS2
NITROGEN_METABOLISM	1.31	5	CA1 CA7 CA4 CA2
HUNTINGTONS_DISEASE	1.29	5	PPARG PPARGC1A
DRUG_METABOLISM_OTHER_ENZYMES	1.27	5	NAT2 UGT1A3 XDH CES2
GLYCOLYSIS_GLUCONEOGENESIS	1.25	6	ALDOB ACSS2 PCK1 ADH1C ADH6
PROXIMAL_TUBULE_BICARBONATE_RECLAMATION	1.24	5	CA4 SLC9A3 CA2 SLC4A4 PCK1
ABC_TRANSPORTERS	1.22	7	ABCG2 ABCB1
GLYCEROPHOSPHOLIPID_METABOLISM	1.21	9	GPD1L PLA2G12B LPGAT1 PLA2G2A CHKA AGPAT4 CHPT1
ALDOSTERONE_REGULATED_SODIUM_REABSORPTION	1.20	5	SCNN1B
DRUG_METABOLISM_CYTOCHROME_P450	1.18	10	FMO5 CYP2B6 UGT1A3 MAOA FMO4 ADH1C ADH6
FATTY_ACID_METABOLISM	1.16	12	EHHADH ADH1C ACADS ADH6 ACAA2 ACOX1 CPT1A CPT2
INSULIN_SIGNALING_PATHWAY	1.15	5	PPARGC1A PTPRF PCK1 PDE3A
VALINE_LEUCINE_AND_ISOLEUCINE_DEGRADATION	1.15	7	HMGCS2 EHHADH ACADS ACAA2
TYROSINE_METABOLISM	1.14	5	MAOA ADH1C DDC ADH6
STEROID_HORMONE_BIOSYNTHESIS	1.13	7	UGT1A3 HSD11B2 HSD17B2 HSD3B2 STS UGT2A3
ARGININE_AND_PROLINE_METABOLISM	1.13	7	MAOA CKB NOS2 OTC ASS1 ALDH18A1
LONG_TERM_DEPRESSION	1.13	5	GNA11 PLA2G12B PRKG2 PLA2G2A
RETINOL_METABOLISM	1.11	7	CYP2B6 UGT1A3 ADH1C RETSAT ADH6
METABOLISM_OF_XENOBIOTICS_BY_CYTOCHROME_P450	1.05	8	CYP2B6 UGT1A3 ADH1C ADH6 CYP2S1 UGT2A3
KEGG_PROGESTERONE_MEDIATED_OOCYTE_MATURATION	1.03	5	RPS6KA6 PDE3A

### Statistical analysis

All data were expressed as mean ± SE. Student’s t test was performed to determine statistical differences between groups. *P* < 0.05 was considered statistically significant.

## Result

### PXR expression in various UC and health samples

**Figure 2:**
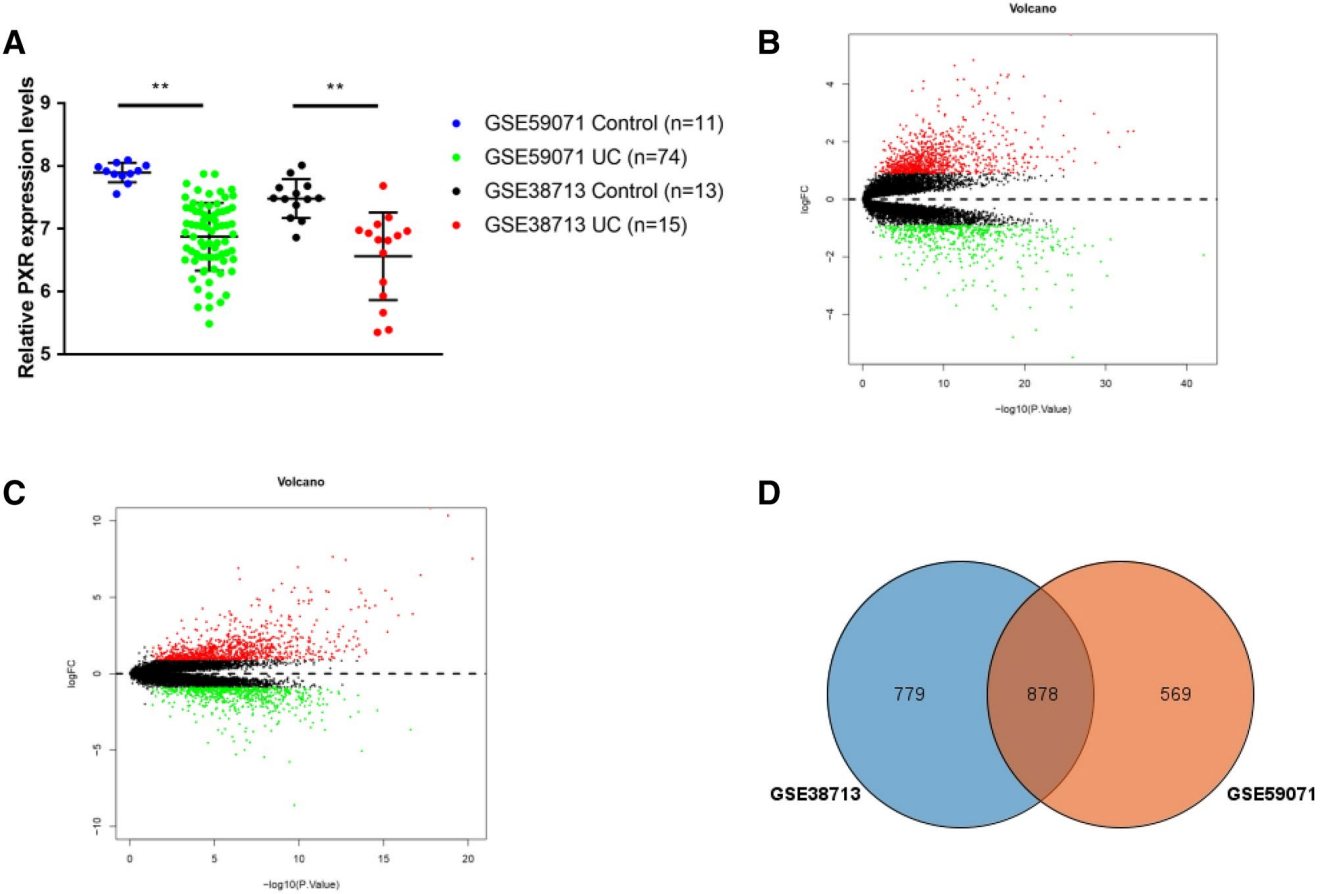
Identification the differentially expressed genes. (**A**) The expression level of PXR in GSE59071 and GSE38713 datasets. **P < 0.01. (**B**, **C**) The volcanoplot of the DEGs between UC patients and control subjects in GSE59071 and GSE38713 datasets. (**D**) The Venn plot for selection of the overlapping DEGs in GSE59071 and GSE38713 datasets, overlapping DEGs = 878.

PXR mRNA expression level in various UC and health samples was assessed. A statistically significant reduction of PXR expression level was found in UC patients compared with control subjects (Fig. 2A,B), indicating that PXR may have a protective role in UC development.

### Identification of DEGs

After data processing, a total 1,447 DEGs (including 549 down-regulated and 898 up-regulated) and 1,657 DEGs (including 689 down-regulated and 968 up-regulated) were identified in GSE59071 and GSE38713 datasets respectively. The volcano plot of DEGs in GSE59071 and GSE38713 was shown in Fig. 2C and D. The 878 overlapping DEGs between the two datasets were obtained by intersection analysis (Fig. 2E).

### WGCNA analysis

**Figure 3:**
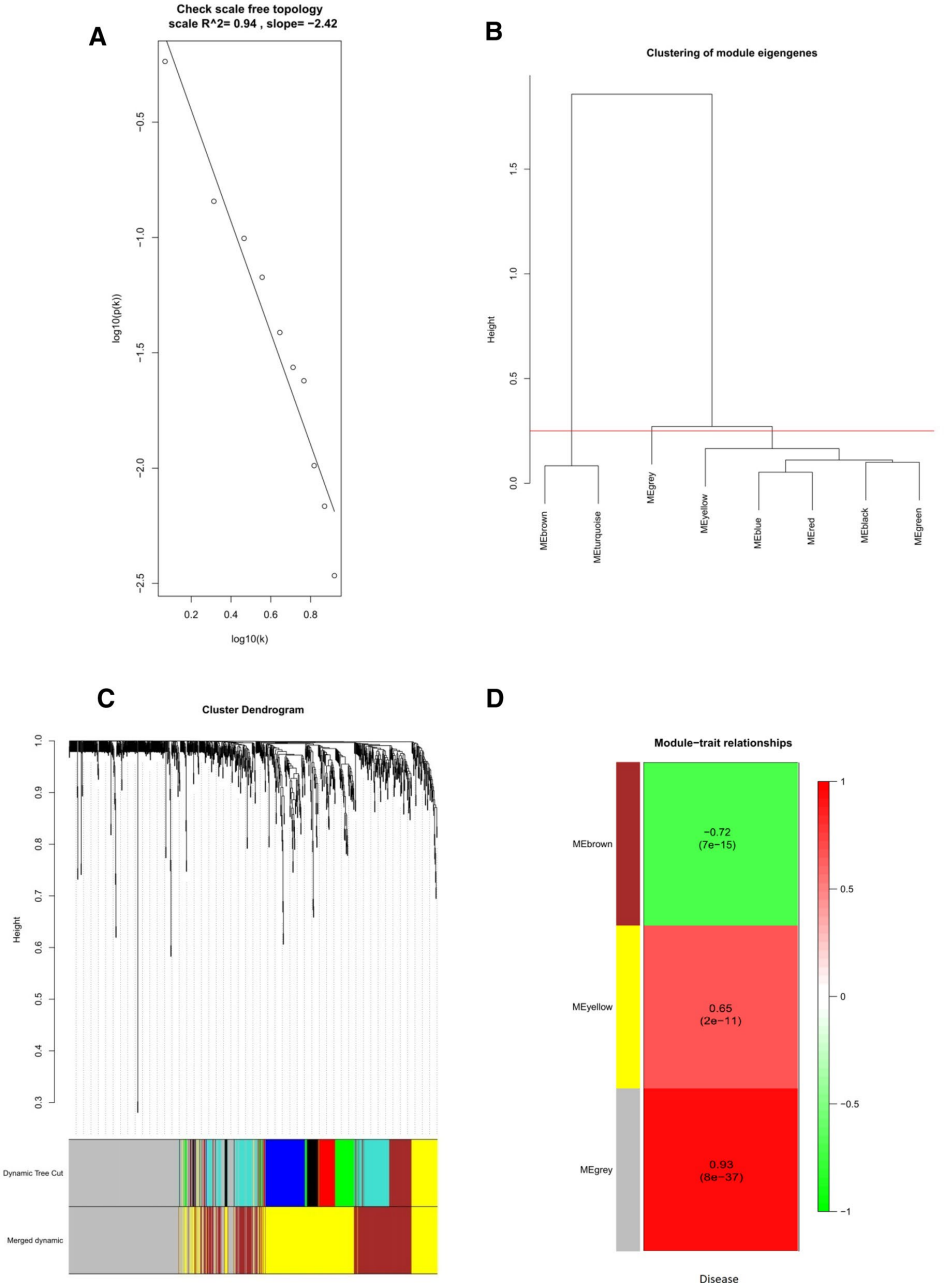
The WGCNA of 878 overlapping DEGs between the UC group and the control group. (**A**) Scale-free networks checking, R^2^ > 0.9. (**B** Module clustering dendrogram. The module is merged with the cutoff value of 0.25. (**C**) Module clustering dendrogram. Each branch in the figure represents one gene, and every color below represents one co-expression module. (**D**) The correlation between each module with UC disease present.

WGCNA analysis was performed using the 878 overlapping DEGs in GSE59071. As shown in Fig. 3A, the value of R^2^ > 0.9, indicating that the scale-free network was successfully constructed. According to the cutoff value of 0.25, DEGs were clustered into two co-expression modules (Fig. 3B,C). The grey module represented a gene set that was not assigned to any of the modules. After combining clinical traits, the brown module (r = −0.72, *P* < 7.0e−15) was negatively correlated with the disease present. The yellow module (r = 0.65, *P* < 2.0e−11) was positively correlated with the disease present (Fig. 3D).

### GSEA associated with PXR

**Figure 4:**
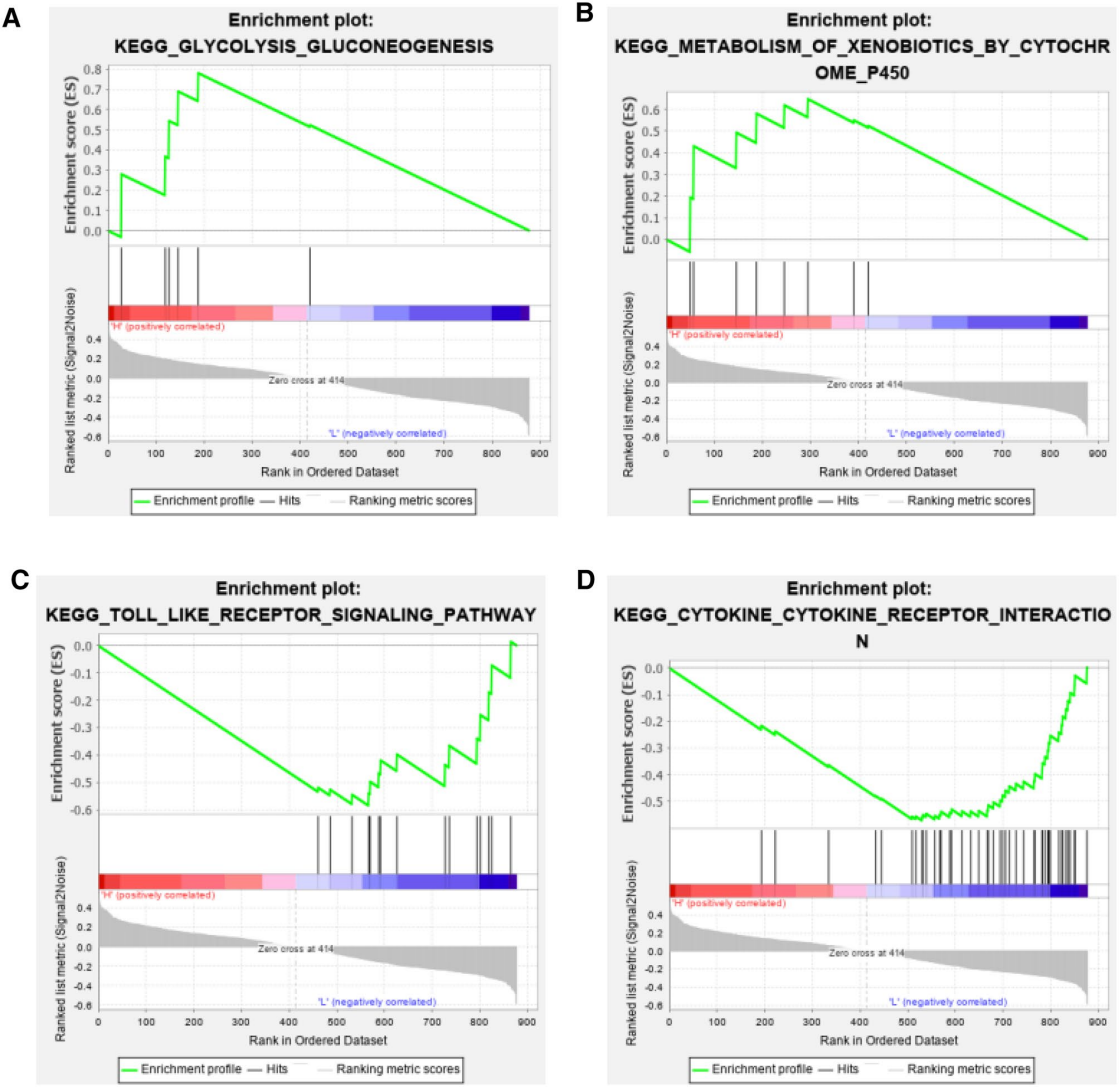
GSEA analysis results. (**A**,**B**) The signal pathway enriched in PXR high expression groups. (**C**,**D**) The signal pathway enriched in PXR low expression groups.

To investigate the possible function and mechanism of PXR in regulating UC, we used the expression matrix of all DEGs for PXR GSEA analysis. The top 20 signal pathways and active genes enriched in high and low expression samples were shown in Table 1 and Table 2 respectively. Among them, signal pathways related to drug and energy metabolism like metabolism of xenobiotics by cytochrome P450 and glycolysis gluconegenesis et al. were enriched in PXR high expression group (Fig. 4A,B), signal pathways related to inflammatory like toll-like receptor signaling pathway, cytokine-cytokine receptor interaction et al. were enriched in PXR low groups (Fig. 4C,D).

**Table 2:** Biological signal pathways and active genes in PXR low expression group by GSEA.

Pathway regulated by PXR	NES	Size	Active genes
PRIMARY_IMMUNODEFICIENCY	− 1.43	7	BTK PTPRC IL7R CD79A
LEUKOCYTE_TRANSENDOTHELIAL_MIGRATION	− 1.39	19	RAC2 NCF4 MSN ICAM1 CDH5 JAM2 NCF2 RASSF5 ITGB2 PECAM1 RHOH MMP9 THY1 CXCR4 VCAM1
VASCULAR_SMOOTH_MUSCLE_CONTRACTION	− 1.29	5	EDNRA CALCRL
CHEMOKINE_SIGNALING_PATHWAY	− 1.21	28	CXCL5 ELMO1 CXCL10 CCL11 CXCR2 CCR1 CCL18 CCL4 CCR7 CXCR4 CXCL9 CXCL6 CCL2 CCL19 CXCL13
SYSTEMIC_LUPUS_ERYTHEMATOSUS	− 1.16	16	C2 HLA-DRA HLA-DMA HLA-DPA1 HLA-DMB CD40 FCGR3B FCGR2C C1R FCGR2A C1S CD86 C3 FCGR2B
B_CELL_RECEPTOR_SIGNALING_PATHWAY	− 1.14	10	PIK3R3 IFITM1 DAPP1 LYN CD81 RAC2 BTK CD79A FCGR2B
CYTOKINE_CYTOKINE_RECEPTOR_INTERACTION	− 1.14	44	CCL3L3 CXCL11 CXCL1 GHR IFNAR2 CD40 IL1B CXCL8 OSMR CSF3R CSF2RB TNFSF13B KDR TNFRSF11B TGFB1 CXCL5 IL10RA IL2RA CXCL10 CCL11 PDGFRB CD27 CXCR2 CCR1 CCL18 CCL4 INHBA CCR7 TNFRSF17 CXCR4 CXCL9 IL7R CXCL6 CCL2 IL24 CCL19 CXCL13
CELL_ADHESION_MOLECULES_CAMS	− 1.14	28	ICAM1 VCAN CDH5 JAM2 ITGB2 PECAM1 ICAM2 SELPLG CD86 PTPRC VCAM1 SELP SELL SELE
FOCAL_ADHESION	− 1.14	22	COL1A1 RAC2 COL3A1 COL4A2 COL1A2 VWF LAMC1 COL6A3 COL5A2 KDR COL4A1 SPP1 THBS2 ITGA5 CAV1 PDGFRB TNC
JAK_STAT_SIGNALING_PATHWAY	− 1.12	12	GHR IFNAR2 OSMR CSF3R CSF2RB IL10RA IL2RA IL7R IL13RA2 IL24
NEUROACTIVE_LIGAND_RECEPTOR_INTERACTION	− 1.06	20	FPR2 C3AR1 FPR1 S1PR1 P2RY13 P2RY8 EDNRA CALCRL
ECM_RECEPTOR_INTERACTION	− 1.05	16	CD44 COL1A1 COL3A1 COL4A2 COL1A2 VWF LAMC1 COL6A3 COL5A2 COL4A1 SPP1 THBS2 ITGA5 TNC
TOLL_LIKE_RECEPTOR_SIGNALING_PATHWAY	− 1.03	15	CXCL11 IFNAR2 CD40 IL1B CXCL8 TLR2 CXCL10 SPP1 CCL4 CD86 CTSK CXCL9 LY96
LYSOSOME	− 1.02	5	CTSH LAPTM5 LAMP3 CTSK
ENDOCYTOSIS	− 1.01	13	RAB31 ASAP1 SMAP2 KDR IL2RA CXCR2 CXCR4
CYTOSOLIC_DNA_SENSING_PATHWAY	− 1.01	7	IL1B AIM2 CXCL10 CCL4 IL33
VIRAL_MYOCARDITIS	− 1.01	13	HLA-DRA HLA-DMA HLA-DPA1 HLA-DMB CD40 RAC2 ICAM1 ITGB2 CAV1 CD86
LEISHMANIA_INFECTION	− 1.00	19	STAT1 HLA-DRA HLA-DMA HLA-DPA1 HLA-DMB IL1B NCF4 TLR2 NCF2 ITGB2 TGFB1 FCGR3B FCGR2C FCGR2A PTGS2 C3
INTESTINAL_IMMUNE_NETWORK_FOR_IGA_PRODUCTION	− 0.99	12	HLA-DRA HLA-DMA HLA-DPA1 HLA-DMB CD40 TNFSF13B TGFB1 CD86 TNFRSF17 CXCR4
PRIMARY_IMMUNODEFICIENCY	− 1.43	7	PIK3R3 LYN RAC2 FCER1G BTK

### Six genes were screened out that can interact and functionally associate with PXR

**Figure 5:**
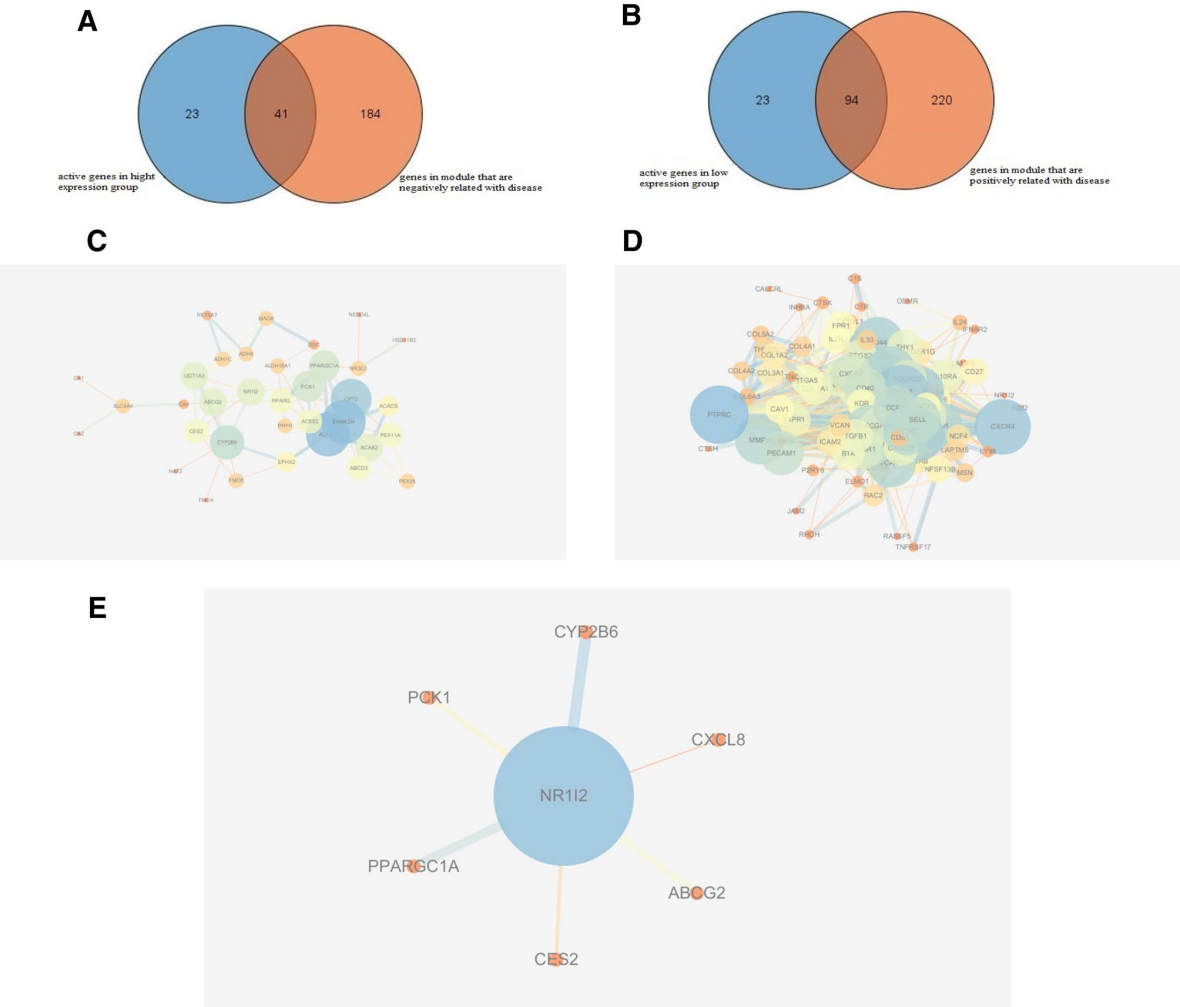
Intersection analysis and protein–protein interaction network with PXR. (**A**) Gene set 1 was constructed by the intersection between the active genes in high expression group after GSEA and the genes in module that are negatively correlated with disease after WGCNA. (**B**) Gene set 2 was constructed by the intersection between the active genes in low expression group after GSEA and the genes in module that are positively correlated with disease after WGCNA. (**C**) PPI-network of 94 overlapping gene in gene set 1. (**D**) PPI-network of 41 overlapping gene in gene set 2. (**E**) CES2, ABCG2, PCK1, PPARGC1A and CYP2B6 from gene set 1 and CXCL8 from gene set 2 were predicted to interact and functionally associate with PXR.

In order to screen the genes that can interact and functionally associate with PXR, intersection and further PPI analysis were performed. First two overlapping gene sets named gene set 1 and gene set 2 were obtained by intersection analysis. The genes in the brown module that are negatively correlated with the disease intersected with the active genes enriched in the signal pathway of the high expression group constructed gene set 1. As shown in Fig. 5A, there are 94 overlapping genes in this set. The genes in the yellow module that are positively related to the disease crossed with the active genes enriched in the signal pathways of low expression group constructed gene set 2. As shown in Fig. 5B, there are 41 overlapping genes in this set. Further PPI analysis with PXR was performed on the two gene sets (Fig. 5C,D). Carboxylesterase 2 (CES2), ATP binding cassette subfamily G member 2 (ABCG2), phosphoenolpyruvate carboxykinase (PCK1), PPARG coactivator 1 alpha (PPARGC1A), cytochrome P450 family 2 subfamily B member 6 (CYP2B6) from gene set 1 and C-X-C Motif chemokine ligand 8 (CXCL8) from gene set 2 that were predicted to interact and functionally associate with PXR were screened out (Fig. 5E). The gene symbol name of these six genes and its signaling pathways they participate in were listed in Table 3.

**Table 3:** Active genes and their corresponding signal pathways they participate in after PPI analysis with PXR.

Gene symbol	Gene name	Signal pathway
PCK1	phosphoenolpyruvate carboxykinase 1	GLUCONEOGENESIS
PPARGC1A	PPARG Coactivator 1 Alpha	INSULIN_PATHWAY
CES2	Carboxylesterase 2	DRUG_METABOLISM_OTHER_ENZYMES
ABCG2	ATP Binding Cassette Subfamily G Member 2	ABC_TRANSPORTERS
CYP2B6	Cytochrome P450 Family 2 Subfamily B Member 6	METABOLISM_OF_XENOBIOTICS_BY_CYTOCHROME_P450
CXCL8	C-X-C Motif Chemokine Ligand 8	TOLL-LIKE_RECEPTOR_SIGNALING_PATHWAY
CYTOKINE-CYTOKINE_RECEPTOR_INTERACTION		

### The six genes were regulated by PXR

**Figure 6:**
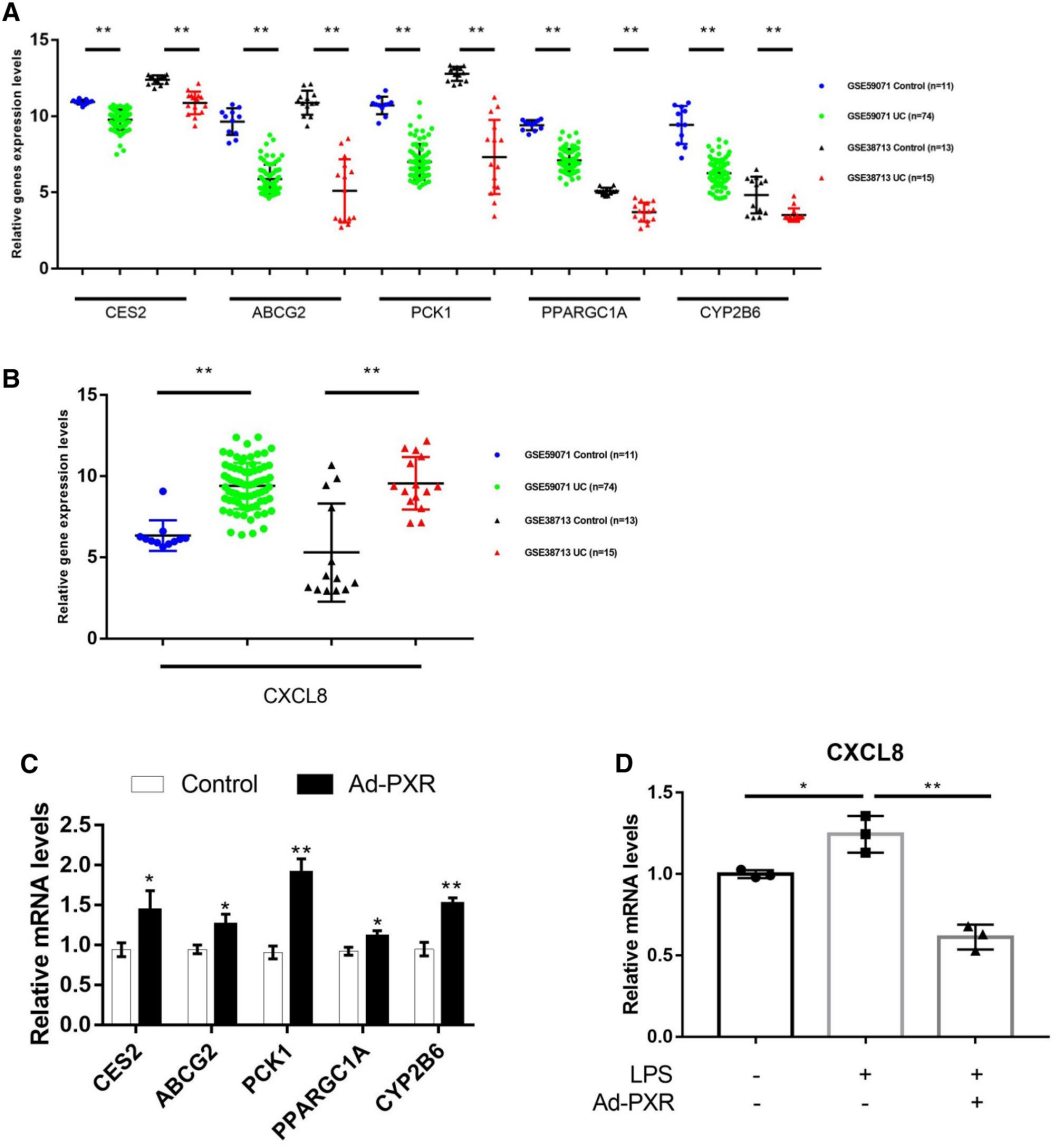
CES2, ABCG2, PCK1, PPARGC1A, CYP2B6 and CXCL8 were regulated by PXR. (**A**) The expression level of CES2, ABCG2, PCK1, PPARGC1A and CYP2B6 in GSE59071 and GSE38713 datasets. (**B**) The expression level of CXCL8 in GSE59071 and GSE38713 datasets. (**C**) PXR increased the expression of CES2, ABCG2, PCK1, PPARGC1A and CYP2B6 in HCT116 cells. HCT116 cells were infected with Ad-PXR or mock infection for 24 h. The mRNA levels of CES2, ABCG2, PCK1, PPARGC1A and CYP2B6 were quantified by RT-qPCR. (**D**) PXR reduced the up-regulated expression of CLX8 induced by LPS. HCT116 cells were infected with Ad-PXR or mock for 24 h before exposure to LPS (1 μg/ml) for 12 h, the mRNA levels of CXCL8 were quantified by RT-qPCR. Data shown are as mean ± S.E. of at least three independent experiments. *P < 0.05 versus control; **P < 0.01 versus control.

The expression level of CES2, ABCG2, PCK1, PPARGC1 and CYP2B6 from gene set 1 were lower in disease than in control group, while the CXCL8 from gene set 2 were higher in disease than in control group (Fig. 6A,B). In order to predict whether the five genes from set 1 are the target genes of PXR, the Pearson’s correlation coefficient between PXR and the five genes expression level was calculated. As shown in supplementary Fig. 1A–J, the Pearson’s correlation coefficient between PXR and the five genes expression level was all greater than 0.5 in two datasets. JASPAR analysis showed that there is at least one PXR binding site on the promoter of these genes. Table 4 showed the predicted sequences that PXR bind on gene promoters with the highest scores. All of these analyses showed CES2, ABCG2, PCK1, PPARGC1A, and CYP2B6 are all possibly PXR target genes. To further verify the bioinformatics analysis results, we examined the expressions levels of CES2, ABCG2, PCK1, PPARGC1A, CYP2B6 and CXCL8 in HCT116 cells. As shown in Fig. 6C and D, overexpression of PXR significantly increased messenger RNA (mRNA) level of CES2, ABCG2, PCK1, PPARGC1A, CYP2B6, and reduced the up-regulated expression of CLX8 induced by lipopolysaccharide (LPS).

**Table 4:** Predicted sequences that PXR bind on the gene promoters listed below with the highest scores.

Gene symbol	Score	Predicted sequence
CES2	8.662	AGGGACTCTGCGCCCTT
ABCG2	7.78554	TTAAAACAATTGAACTC
PCK1	8.51	GTGCCCCGGCCAACCTT
PPARGC1A	7.97	TTAAACCCAATATACTT
CYP2B6	10.3884	GTGATCTCTGTGACCTA

## Discussion

More and more documents showed that the activation of PXR has a protective role on UC. Antibiotic rifaximin, a PXR agonist was used clinically for the treatment of UC^9^. In order to deeply explore the molecular mechanism of PXR on UC regulation, integrated bioinformatics methods were used in this study.

We first used two GEO database (GSE59071 and GSE38713) to statistically analyze the expression level of PXR in the health and UC groups. The results showed that the expression level of PXR was significantly lower in UC compared to health groups, which was consistent with Langmann and other authors’ reports^28,29^, indicating PXR is negatively related to the disease. WGCNA can cluster genes with co-expression patterns into the same module and associate the modules with clinical traits. After WGCNA analysis by using 878 DEGs, the brown module that is positively related to disease and yellow module that is negatively related to the disease was identified. Then single PXR gene of GSEA was performed to obtain the signal pathway enriched in PXR high expression or low expression group. As PXR expression level is low in UC disease and has a protective role on UC, PXR may protect UC by positively regulating the signal pathway enriched in the high expression group or negatively regulating the signal pathway enriched in the low expression group. Based on analysis of WGCNA and GSEA, we speculated that if PXR can interact or functionally associated with genes that are both active in the high expression group and negatively related to diseases, then PXR may inhibit UC by promoting these genes expression. Similarly, if PXR can interact or functionally associated with genes that are both active in the low expression group and positively related to diseases, then PXR may play a role in protecting UC by inhibiting these gene expressions. As protein interaction analysis based on the string database includes not only the direct (physical) interaction but also indirect (functional) association, the PPI network with PXR was performed by using these two gene sets (gene set 1 and gene set 2). Finally, five genes (CES2, ABCG2, PCK1, PPARGC1A, CYP2B6) from set 1 and one gene (CXCL8) from set 2 were screened out (Fig. 5E). In this way, PXR can play a protective effect on UC by promoting CES2, ABCG2, PCK1, PPARGC1A, CYP2B6 expression, or inhibiting CXCL8 gene expression. Furthermore, the results of RT-qPCR further confirmed that PXR indeed increased the expression level of CES2, ABCG2, PCK1, PPARGC1A, CYP2B6, and inhibited the expression of CXCL8 induced by LPS.

The five genes (CES2, ABCG2, PCK1, PPARGC1A, CYP2B6) are all distributed in pathways related to metabolism, such as glucose and lipid metabolism and drug metabolism. PCK1 is a key enzyme regulating liver gluconeogenesis. It has been reported that activation of PXR in mice can inhibit PCK1 expression by inhibiting FOXO1 (forkhead box protein O1), the main transcription factor of PCK1, or by competitively binding to peroxisome proliferator-activated receptor-gamma coactivator 1α (PPARGC1A) with hepatocyte nuclear factor 4 alpha (HNF4α)^30^. However, the regulation of PCK1 expression in human showed the opposite result. PXR up-regulates the expression of PCK1 through the unique human PXR-SGK2 signaling pathway^31^. Our results showed that the expression level of PCK1 in UC patient samples is down-regulated as PXR, and Pearson's correlation coefficient and JASPAR analysis both showed that PCK1 maybe the target gene of PXR. Therefore, it is speculated that PXR may directly initiate PCK1 transcription and up-regulates its expression to promote gluconeogenesis in colon tissue. Further literature mining suggested that UC disease showed lower nutritional levels and excessive energy consumption^32^. At the same time, lipid metabolism and tricarboxylic acid cycle metabolism levels in UC disease decreased, resulting in the loss of energy homeostasis^33–35^. Studies have reported that in addition to liver and kidney, gluconeogenesis genes are indeed expressed in intestinal tissues^36,37^, and intestinal gluconeogenesis can promote glucose and energy balance^36^. Therefore, we speculated that PXR may protect UC by promoting the expression of PCK1 and increasing the level of gluconeogenesis in intestinal tissues.

Studies have shown that PPARGC1A is highly expressed in intestinal epithelial cells, but its expression is low in UC diseases^38^, which is consistent with the expression level of the GEO dataset (GSE59071 and GSE38713). Lack of PPARGC1A in mouse intestinal epithelial cells aggravates the occurrence of colitis^38^, showing the protective effect of PPARGC1A on diseases. Studies have reported that the dysfunction of mitochondrial oxidative phosphorylation plays an important role in the occurrence of UC^39^. Literature mining results showed that PPARGC1A can maintain mitochondrial functions, such as inhibiting oxidative stress, controlling the balance of ROS levels and inhibiting inflammation by promoting mitochondrial biogenesis in intestinal epithelial cells^38^. What’s more, mitochondria are of great significance in maintaining the function of the electron transport chain and generating ATP energy. UC disease has low energy characteristics^32^, which is obviously not helpful for the formation of tight junction that requires energy in intestinal epithelial cells^40^. Damage to tight junction will lead to the invasion of pathogens and aggravate the occurrence of UC^41^. Therefore, PPARGC1A may ensure the integrity of the physical barrier of intestinal epithelial cells through mitochondrial biogenesis^38^. Combined with the results of our study, we speculated that PXR could play an important protective role in mitochondrial biogenesis by up-regulating the expression of PPARGC1A.

It is widely believed that detoxification and biotransformation play an important role in protecting intestinal epithelial cells^42^. The deletion of detoxification enzyme genes is an important event leading to the initiation and development of UC disease^43^. GEO database data shows that CES2, CYP2B6, and ABCG2 detoxification genes are down-regulated in disease samples (Fig. 6 A,B). As the main transcription factor of detoxification genes, we speculated that PXR can play an anti-UC effect by regulating expression of detoxification genes like CES2, CYP2B6, and ABCG2 in intestinal tissue. Although the literature has shown that PXR can regulate the expression of CYP2B6 and ABCG2, the regulation role is not occurred in the intestinal tissues^44,45^. Therefore, our results revealed new targets for PXR to regulate detoxification genes in intestinal tissues.

The abnormal immune response is the main pathological change when UC occurs, mucosal inflammation caused by immune cell infiltration is the main histopathological feature of UC^28,46,47^. CXCL8 is a member of the CXC chemokine family. It can induce neutrophils to accumulate to the diseased colon mucosa through its receptors, causing tissue infiltration^47,48^, stimulating neutrophils degranulation, and inducing respiratory bursts. The resulting large amount of toxic substances trigger an intestinal inflammatory response, leading to the destruction of the intestinal barrier and tissue damage^49^. As a pattern recognition receptor for pathogens, TLR (Toll-like receptor) can sense the stimulation of external pathogens and activate NF-κB to produce inflammatory factors and trigger an inflammatory response. Studies have reported that TLR4 of intestinal epithelial cells was stimulated by pathogenic bacteria to up-regulate the expression of CXCL8 via NF-κB^50^, thereby increasing the infiltration of neutrophils, aggravating inflammation, and causing damage to intestinal tissue^51^. In addition, the necrosis of intestinal epithelial cells and the subsequent release of DAMPs (damage-associated molecular patterns) caused by intestinal tissue damage can also further activate TLR2 and trigger the occurrence of inflammation^52–54^. Numerous studies have shown that PXR inhibits the up-regulated expression of CXCL8 by inhibiting NF-κB^50,55^. Our combined analysis of WGCNA and PXR GSEA showed that PXR can protect UC by inhibiting the up-regulated expression of CXCL8.

## Conclusion

**Figure 7:**
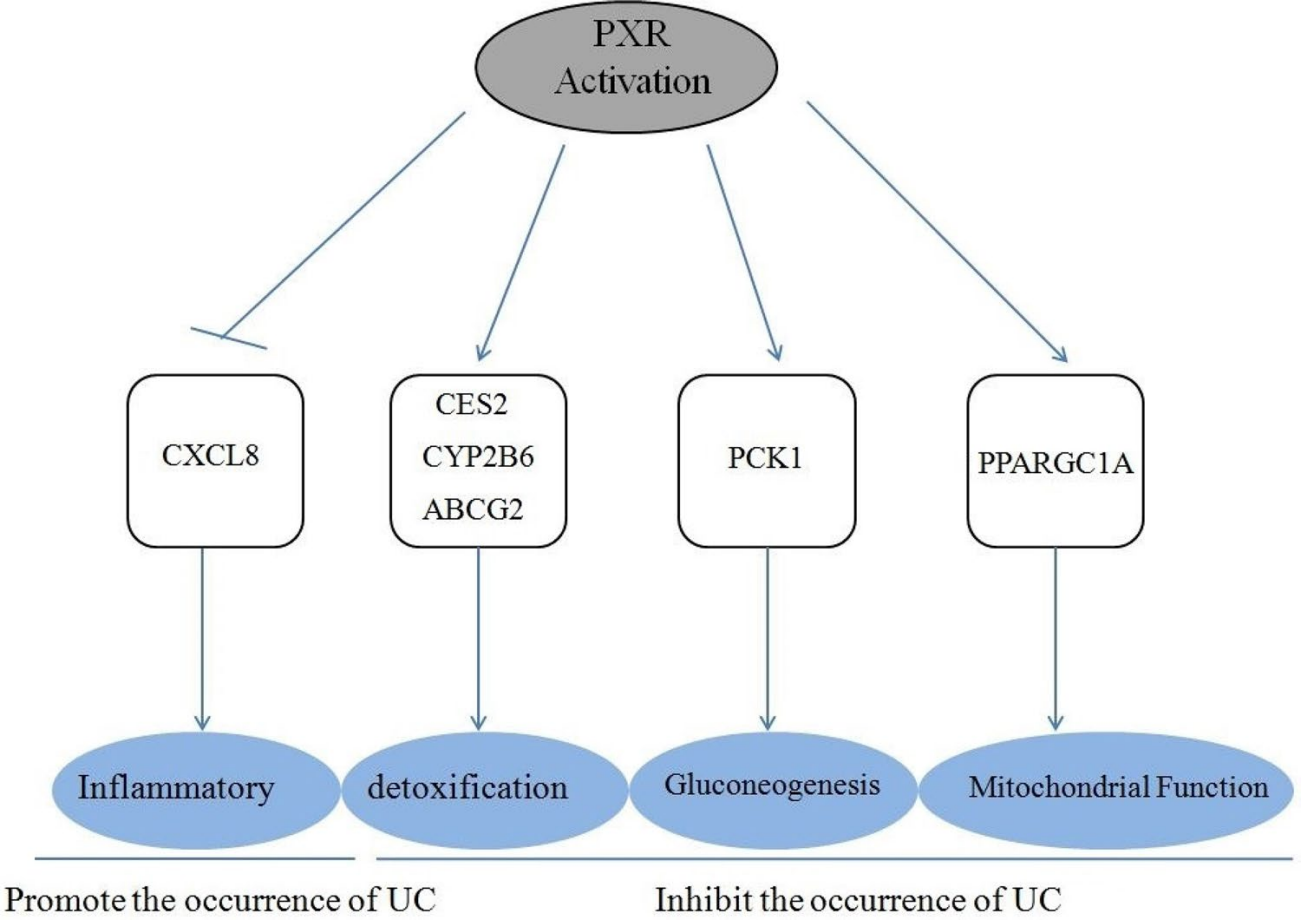
Protection role of PXR on UC.

After integrated bioinformatics analysis and RT-qPCR test, we thought that PXR may protect UC by promoting intestinal gluconeogenesis, maintaining the intestinal mitochondrial function, promoting detoxification through upregulation of CES2, ABCG2, PCK1, PPARGC1A, CYP2B6 gene expression, and inhibiting the expression of the inflammatory factor CXCL8 (Fig. 7).

## Supplementary Information

Supplementary Figure 1.

Supplementary Information

## Data Availability

All authors make sure that all data and materials support published claims and comply with field standards.
