# Supplementary material for: Bioinformatis analysis reveals possible molecular mechanism of PXR on regulating ulcerative colitis
Source: Sci Rep. 2021 Mar 8;11:5428. doi: 10.1038/s41598-021-83742-8 (PMC7940411; doi:10.1038/s41598-021-83742-8)
Supplement: Supplementary file 1 — Supplementary Figure 1. [file 41598_2021_83742_MOESM1_ESM.pdf]

Bioinformatis analysis reveals possible molecular mechanism of PXR on regulating inflammatory bowel disease

Hanze Guo[1,2], Yan Chi[1]*, Naiyu Chi[2]*,

1. College of Life Sciences, Liaoning Normal University, Dalian116081, China
2. College of Life Science and Technology, Dalian University, Dalian 116622, China

*Corresponding author:

Yan Chi, Email: chiyan@lnnu.edu.cn

Naiyu Chi, Email: cny7566@126.com

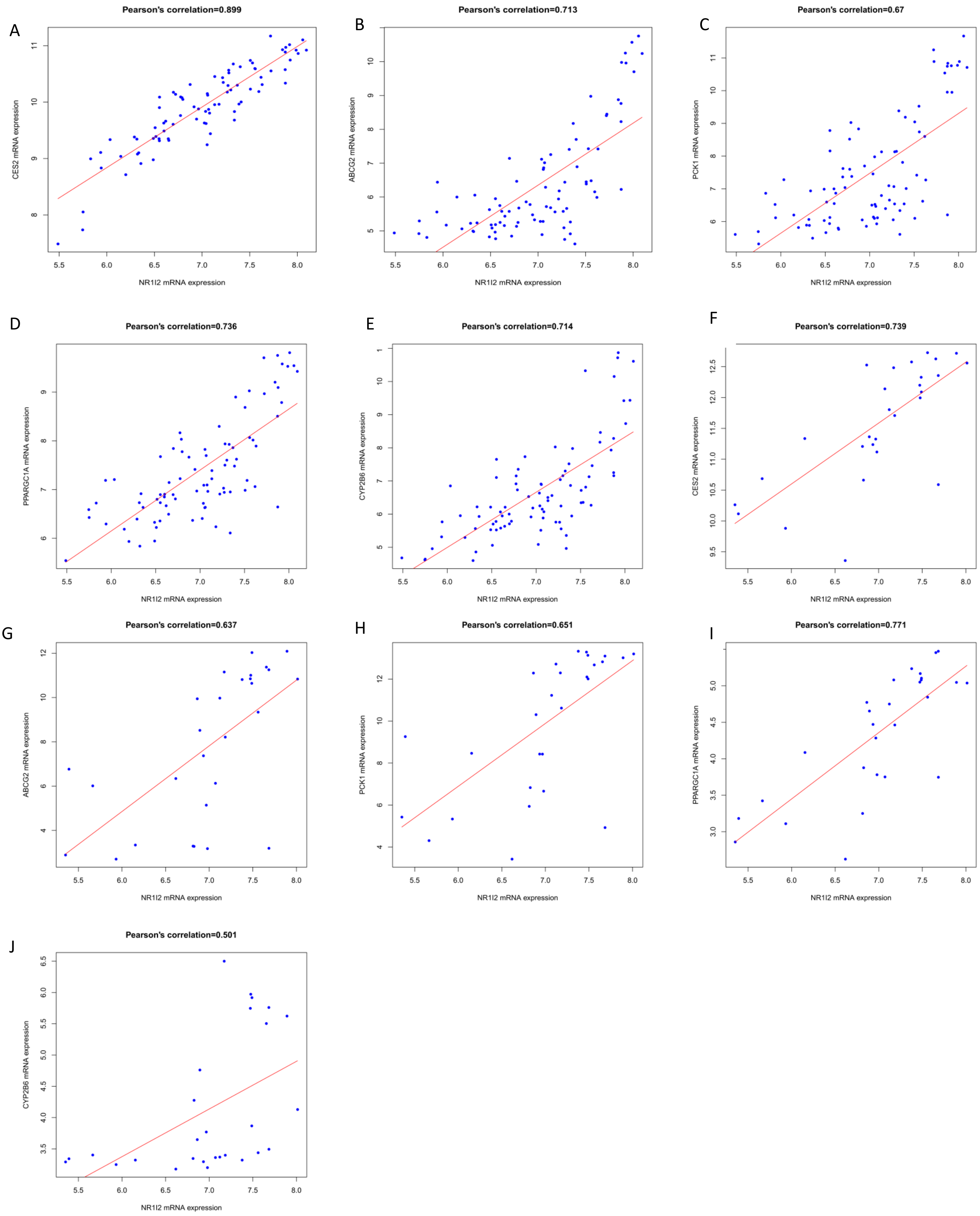
A
Pearson's correlation=0.899
CES2 mRNA expression
NR1I2 mRNA expression
B
Pearson's correlation=0.713
ABCG2 mRNA expression
NR1I2 mRNA expression
C
Pearson's correlation=0.67
PCK1 mRNA expression
NR1I2 mRNA expression
D
Pearson's correlation=0.736
PPARGC1A mRNA expression
NR1I2 mRNA expression
E
Pearson's correlation=0.714
CYP2B6 mRNA expression
NR1I2 mRNA expression
F
Pearson's correlation=0.739
CES2 mRNA expression
NR1I2 mRNA expression
G
Pearson's correlation=0.637
ABCG2 mRNA expression
NR1I2 mRNA expression
H
Pearson's correlation=0.651
PCK1 mRNA expression
NR1I2 mRNA expression
I
Pearson's correlation=0.771
PPARGC1A mRNA expression
NR1I2 mRNA expression
J
Pearson's correlation=0.501
CYP2B6 mRNA expression
NR1I2 mRNA expression
