# Supplementary material for: Bioinformatis analysis reveals possible molecular mechanism of PXR on regulating ulcerative colitis
Source: Sci Rep. 2021 Mar 8;11:5428. doi: 10.1038/s41598-021-83742-8 (PMC7940411; doi:10.1038/s41598-021-83742-8)
Supplement: Supplementary file 2 — Supplementary Information [file 41598_2021_83742_MOESM2_ESM.docx]

Bioinformatis analysis reveals possible molecular mechanism of PXR on regulating Ulcerative Colitis

Hanze Guo^1,2^, Yan Chi^1^*, Naiyu Chi^2^*,

1. College of Life Sciences, Liaoning Normal University, Dalian116081, China
2. College of Life Science and Technology, Dalian University, Dalian 116622, China

*Corresponding author:

Yan Chi, Email: chiyan@lnnu.edu.cn

Naiyu Chi, Email: cny7566@126.com

[Supplementary](https://www.nature.com/srep/author-instructions/submission-guidelines#supplementary-info) Table 1: Primer sequences

| **Gene** | **Primer Sequence (5’→3’)** |
| --- | --- |
| CES2 | Forward GTAGCACATTTTCAGTGTTCC |
|  | Reverse GTAGTTGCCCCCAAAGAA |
| CYP2B6 | Forward GGAGGAGCGGATTCAGGAGGAG |
|  | Reverse AGCAGATGATGTTGGCGGTAATGG |
| PPARGC1A | Forward GGGAGAGGCAGAGGCAGAAGG |
|  | Reverse CTGTCCGTGTTGTGTCAGGTCTG |
| ABCG2 | Forward TATCAATGGGATCATGAAACCTGG |
|  | Reverse GCGGTGCTCCATTTATCAGAAC |
| PCK1 | Forward CTGGATGAAGTTTGACGCAC |
|  | Reverse GGTCTTCACTGAAGTCCCAG |
| CXCL8 | Forward CCCTCTGCACCCAGTTTTCCTTG |
|  | Reverse GGACCACACTGCGCCAACAC |
| GAPDH | Forward AACAGCCTCAAGATCATCAGC |
|  | Reverse GGATGATGTTCTGGAGAGCC |
